# False lateralization of scalp EEG and semiology in cavernous malformation-associated temporal lobe epilepsy: A case report

**DOI:** 10.1016/j.heliyon.2023.e18237

**Published:** 2023-07-13

**Authors:** Tomohiro Nakamura, Keisuke Hatano, Keishiro Sato, Hideo Enoki, Ayataka Fujimoto

**Affiliations:** a Comprehensive Epilepsy Center, Seirei Hamamatsu General Hospital, 2-12-12 Sumiyoshi, Nakaku, Hamamatsu, Shizuoka, 430-8558, Japan; b Department of Neurosurgery, Seirei Hamamatsu General Hospital, 2-12-12 Sumiyoshi, Nakaku, Hamamatsu, Shizuoka, 430-8558, Japan; c Department of Pediatrics, Kawasaki Medical School Hospital, 577 Matsushima, Kurashiki, Okayama, 701-0192, Japan

**Keywords:** False lateralization, Cavernous malformation, Temporal lobe epilepsy, Epilepsy surgery, Case report

## Abstract

**Background:**

Several cases of temporal lobe epilepsy (TLE) showing false lateralization of ictal scalp electroencephalography (EEG) have been reported. However, TLE with cavernous malformation indicating false lateralization of both ictal scalp EEG and semiology as in the present case is rare. The aim of this report is to call attention to avoiding overestimation of ictal scalp EEG findings in epilepsy patients with cavernous malformation.

**Case report:**

A 25-year-old man without any medical history suffered from seizures for a year despite appropriate anti-epileptic medication. Magnetic resonance imaging (MRI) revealed cavernous malformation in the left amygdala. The seizure type was brief impaired consciousness with left dystonic posturing, preceded by a sensation of blood rushing to the head. Long-term video EEG with scalp electrodes showed periodic sharp waves beginning from the right temporal area during seizures. Although both semiology and ictal scalp EEG indicated right TLE, intracranial EEG revealed the onset of low-voltage fast activity from the left hippocampus near the cavernous malformation. This patient therefore underwent removal of cavernous malformation and left amygdala, and achieved freedom from seizures postoperatively.

**Conclusion:**

We reinforce the importance of performing intracranial EEG for cavernous malformation-associated epilepsy when discrepancies between scalp EEG and MRI are evident.

## Highlights

• Scalp ictal electroencephalogram (EEG) showed false lateralization.
• Semiology sometimes indicates false lateralization.
• Intracranial EEG may be important in cavernous malformation-associated epilepsy.

## 1 Introduction

Resection of epileptogenic tissue has been widely performed as a curative treatment for patients with drug-resistant epilepsy. To identify the epileptic focus, a variety of assessments are performed prior to focus resections, including video electroencephalography (EEG), imaging tests such as magnetic resonance imaging (MRI), positron emission tomography (PET), single-photon emission computed tomography, magnetoencephalography, and neuropsychological tests. Intracranial EEG may be skipped in patients with concordant findings on all of these examinations [1]. However, discordance between these tests warrants further inspections, such as intracranial EEG.

Ictal EEG findings from long-term video scalp EEG tend to be emphasized when deciding on foci [2,3], and cases with ictal scalp EEG that falsely indicates laterality of the seizure focus have been reported as “false lateralization” [4]. Many case reports have described false lateralization with severe hippocampal sclerosis or cortical atrophy, because these foci generate ictal waves that are too small to be detected by scalp EEG [5,6]. On the other hand, only two case reports have described false lateralization of scalp EEG with cavernous malformation [4,7]. However, whether it was scalp EEG or cavernous malformation localization that incorrectly lateralized the seizure onset remained unclear because intracranial EEG was not performed in either of those two cases.

This report describes an extremely rare case in which both semiology and scalp EEG falsely indicated the laterality of the seizure onset zone, despite a lack of hippocampal sclerosis or cortical atrophy. Based on this case report, we would like to suggest avoiding an overvaluation of ictal scalp EEG and semiology in cavernous malformation-associated temporal lobe epilepsy.

## 2 Case report

**Fig. 1:**
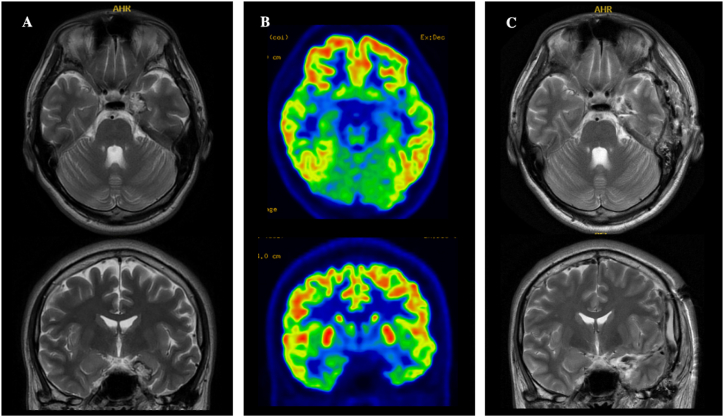
Axial- and coronal-view MRI and FDG-PET. T2-weighted MRI reveals a popcorn lesion with central hyperintensity and a peripheral hypointense rim in the left amygdala (A). This finding is compatible with cavernous malformation. FDG-PET shows slight glucose hypometabolism in the left mesial temporal (B), consistent with the lesion on MRI. MRI at 3 days after the removal surgery shows complete resection of cavernous malformation (C).

A right-handed, 25-year-old man without any medical history such as febrile seizures, head trauma or intellectual disability had presented with focal impaired awareness seizure (FIAS) with unnatural posturing of the left upper limb at 24 years old. Perampanel (4 mg/day) was unable to control seizures. MRI revealed a small “popcorn” lesion of mixed signal intensity with hypointense rim in the left amygdala on T2-weighted imaging (Fig. 1), representing a finding consistent with cavernous malformation. PET of the brain using fluorodeoxyglucose (FDG) showed low FDG uptake in the left medial temporal lobe, representing the same site as the lesion on MRI. However, this FDG-PET finding was unclear (Fig. 1). Neuropsychological assessment revealed slight impairment of language function (Wechsler Adult Intelligence Scale-fourth edition: full scale, 101; verbal comprehension, 83; perpetual reasoning, 124; working memory, 100; processing speed, 90; Wechsler Memory Scale-revised: verbal memory, 80; visual memory, 94; general memory, 81; attention/concentration, 103; delayed recall, 75). The language-dominant hemisphere was the left side according to the Wada test. These findings suggested the epileptic focus was on the left side.

**Fig. 2:**
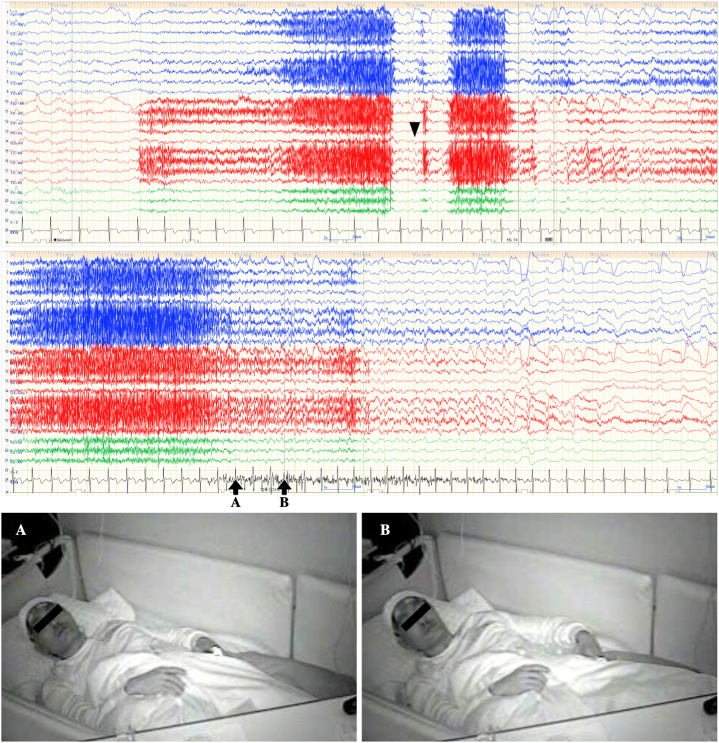
Long-term video scalp EEG during a seizure (amplitude, 10 μV; time constant, 0.1; high frequency filter, 60 Hz; average referential montage). Left, right, and central EEG findings are represented in blue, red, and green, respectively. Periodic sharp waves start in the right temporal area (arrowhead). The left upper extremity begins to move (arrow A, image A) and shows dystonic posturing (arrow B, image B). (For interpretation of the references to colour in this figure legend, the reader is referred to the Web version of this article.)

Long-term (48-h) video EEG with scalp electrodes was performed after discontinuing perampanel and 5 seizures were recorded. The seizure began with a “head rush” sensation, followed by impaired awareness with unusual posturing of the left upper extremity. One of the 5 seizures showed a left dystonic posture (Fig. 2), although 2 seizures showed left tonic posturing with finger opening and the other 2 seizures were not visible on video. After approximately 10 seconds, the patient recovered consciousness and unnatural posturing remitted. No oroalimentary or manual automatisms were observed throughout the examination. Ictal scalp EEG started with periodic sharp waves in the right temporal area, gradually increasing in amplitude (Fig. 2). During interictal periods, sharp waves were observed only in the right temporal region, while no interictal epileptiform discharges (IEDs) were observed in the left temporal region. Because of the left dystonic posturing and scalp EEG findings as described above, the seizure onset zone was suspected to be the right temporal lobe, although the tumor was present on the left side.

**Fig. 3:**
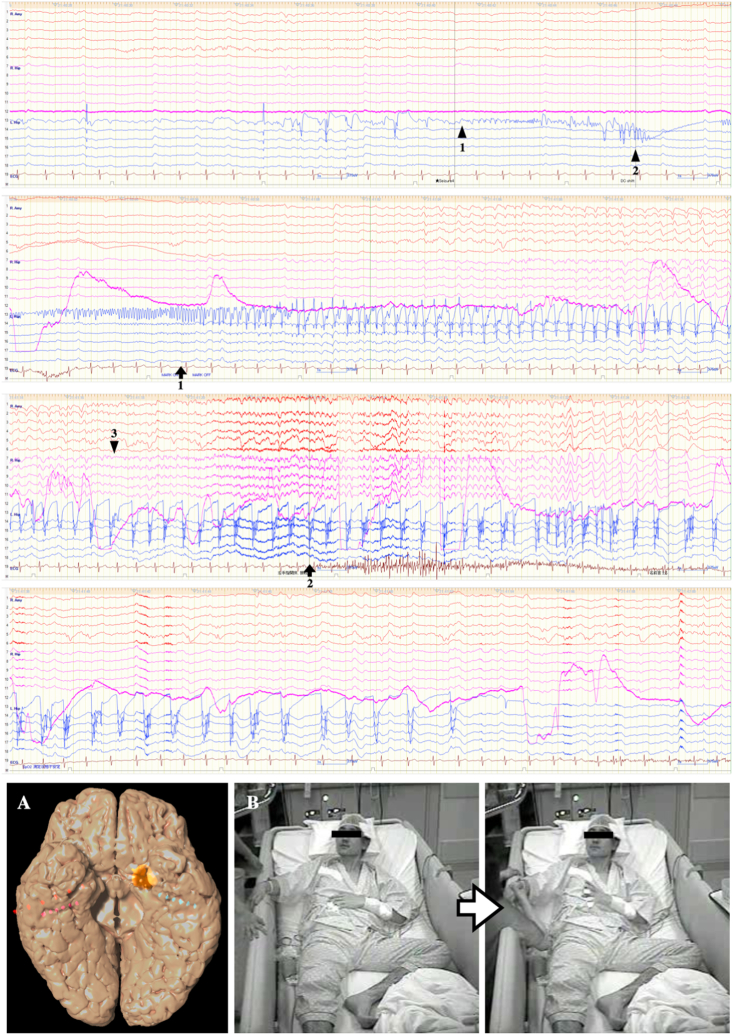
Intracranial EEG during seizure (amplitude, 75 μV; time constant, 2.0 s; high frequency filter, 120 Hz). SEEG leads were inserted into the right amygdala and bilateral hippocampus (image A). EEG findings for the right amygdala, right hippocampus, and left hippocampus are represented in red, pink, and blue, respectively. LVFA (arrowhead 1) and DC shift (arrowhead 2) appeared in the left hippocampus. After onset of these EEG findings, this patient pushed the mark-on button signaling seizure onset with a sense of blood rushing to the head (arrow 1). Onset of LVFA in the right hippocampus is shown 35 seconds after LVFA onset in the left hippocampus (arrowhead 3). After the appearance of LVFA in the right hippocampus, tonic posturing of the left hand with finger opening is apparent (arrow 2, image B). (For interpretation of the references to colour in this figure legend, the reader is referred to the Web version of this article.)

Due to the discordance between imaging and scalp EEG, we conducted stereotactic electroencephalography (SEEG) with three targets, in bilateral hippocampi and the right amygdala, before focus resection surgery (Fig. 3). In our institution, depth electrodes (Unique Medical Co., Komae, Japan) were implanted by neuronavigation-guided frameless SEEG [8]. We used the VarioGuide system (Brainlab AG, Munich, Germany) under the Curve™ neuronavigation system (Brainlab AG). No complications such as intracerebral hemorrhage, cerebral edema, infection, and electrode misplacement were observed. Intracranial EEG displayed low-voltage fast activity (LVFA) and direct current (DC) shift starting from the left hippocampus near the tumor before he felt the head rush sensation and pushed the mark-on button signaling seizure onset. The ictal wave slowly increased in amplitude, decreased in frequency, and spread into the right hippocampus earlier than into the left temporal neocortex. After the propagation of LVFA to the right hippocampus, the patient experienced impaired awareness and tonic posturing was apparent with the left upper extremity. During interictal periods, spike waves were frequently observed in the left hippocampus. Although IEDs were also found in the right hippocampus, the frequency of IEDs in the right hippocampus was less than one-tenth of that in the left. Based on these findings from intracranial EEG, we considered that the seizure onset zone was located in the left temporal lobe causing focal aware seizure (FAS) and that ictal waves spread rapidly to the right hippocampus and caused FIAS with left-sided tonic or dystonic posturing. The right temporal lobe was therefore assumed to be the symptomatogenic zone for FIAS.

We eventually diagnosed this patient with left cavernous malformation-associated temporal lobe epilepsy (TLE) and performed resection of the cavernous malformation and left amygdala under a transcortical approach. MRI after surgery confirmed complete removal of the cavernous malformation (Fig. 1). The pathological result was benign hemangioma and no abnormalities were evident in the temporal lobe or amygdala. No postoperative complications were observed, including memory disorders and aphasia. As of the time of writing, 3 months postoperatively, the patient has achieved freedom from seizures.

## 3 Discussion

The current case involved cavernous malformation-associated temporal epilepsy, with false lateralization from both scalp EEG and semiology. The results of intracranial EEG confirmed that laterality of the epileptic focus was misguided by scalp EEG, suggesting that LVFA originating from the left medial temporal area near the cavernous malformation propagated rapidly to the contralateral hippocampus and spread throughout the right temporal region earlier than the left side. Although three reports have described the side of ictal onset in scalp EEG contralateral to cavernous malformation [3,4,7], to the best of our knowledge, the present case represents the first report that intracranial EEG identified false lateralization of scalp EEG and true lateralization of MRI findings to the seizure onset zone. Moreover, another notable point of this case was that semiology, particularly in the form of the left dystonic posture, also led to false lateralization of seizure onset. We reiterate the importance of performing intracranial EEG rather than relying solely on ictal findings of scalp EEG and semiology.

Although long-term video scalp EEG is an inevitable non-invasive examination before focal resection surgeries [2], several authors have documented cases with false lateralization from scalp EEG [4]. Previous reports have attributed false lateralization to severe hippocampal sclerosis (burned-out hippocampus) or cortical atrophy [5,6,9]. The voltage of ictal waves in these pathologies was considered too low to be investigated by scalp EEG, resulting in misinterpretation of findings as indicating contralateral seizure onset. Mintzer et al. described scalp EEG displaying seizure onset falsely on the side of the intact hippocampus in 4.7% of temporal lobe epilepsies with severe hippocampal sclerosis [5]. Rapid spread to the contralateral side through functional commissural connections such as the hippocampal commissure is another hypothesis for the mechanism of false lateralization [4,10]. This theory would apply to the current case because intracranial EEG showed ictal waves spreading earlier to the contralateral hippocampus than to the ipsilateral temporal neocortex. On the other hand, unlike previous reports, severe hippocampal sclerosis and cortical atrophy were not seen in this case. Although the reason why ictal waves were less likely to spread ipsilaterally remains unclear, we speculated that cavernous malformation in the left amygdala itself may have interrupted the propagation of ictal waves to the left temporal neocortex.

Unilateral dystonic posturing is considered a key sign for judging lateralization of the seizure focus, since the probability that the focus of seizure onset is located contralateral to the side of dystonic posturing is reported as 92–100% [[11], [12], [13], [14]]. Dystonic posture is an unnatural posture of the distal upper limb and is attributed to propagation of ictal waves to the ipsilateral basal ganglia [15,16]. The typical posture involves flexion of the wrist and metacarpophalangeal joints, extension of the fingers, and rotation of the forearm [11]. However, in the present case, dystonic posture was found on the same side as the seizure focus. The poor reproducibility of left dystonic postures (1 dystonic posture, 2 tonic postures with finger opening, and 2 unable to be observed, out of 5 seizures during long-term video EEG) may have been associated with the false lateralizing signs of semiology in this case. We presume that the mechanism by which this patient did not exhibit right dystonic posturing may have been disruption of connectivity extending from the left amygdala to the left basal ganglia [17] because cavernous malformation widely occupied the left amygdala.

Patients with a more than 75–90% probability of having IEDs unilaterally are more likely to have the seizure foci on the side with more IEDs and achieve a seizure-free state after focus resection surgery [18,19]. IED findings in scalp EEG of this case therefore also suggested right temporal lobe epilepsy, since all IEDs were in the right temporal region. Temporal lobe epilepsy with MRI lesions such as hippocampal sclerosis has been reported to have a 6.3–9.5% chance of having more IEDs on the healthy side than on the pathological side [20,21]. Localization of IEDs, as well as the ictal wave and semiology, opposite to the seizure onset zone, as in the present case, seems extremely rare. The false lateralization of IEDs in this case may have been caused by the opposite orientation of spike dipoles rather than by spike propagation from the left to the right hippocampus. Since intracranial EEG revealed that more than 90% of IEDs appeared in the left hippocampus, the dipole of spikes generated in the left hippocampus may have been directed to the right, resulting in right temporal IEDs on scalp EEG.

There were some limitations in this case report. First, we cannot discuss magnetoencephalography or single photon emission computed tomography, as we did not use these examinations. Second, we did not perform simultaneous scalp and intracranial EEG. Proving the mechanism of the IED false lateralization described above would require simultaneous scalp and intracranial EEG.

Although the ictal and interictal scalp EEG and semiology strongly suggested right TLE, the intracranial EEG confirmed left TLE in this patient. The seizure onset zone was in the vicinity of the cavernous malformation, such as the left hippocampus, but early propagation of ictal waves to the right hippocampus caused left dystonic posture and false lateralizing findings of scalp EEG in this case. Although ictal EEG findings are reportedly more credible than imaging [3], intracranial EEG appears necessary to confirm the seizure focus in patients with cavernous malformation-associated epilepsy if inconsistency is apparent between scalp EEG and results from imaging. Reports about false lateralization in cavernous malformation are rare. Therefore, additional case accumulation and further research are required to reveal the frequency and features of false lateralization in cavernous malformation-associated epilepsy.

## Ethics approval and consent to participate

No ethical approval was needed because this was an anonymous case report. The patient signed a consent form for publication. We adhered to the Declaration of Helsinki.

## Consent for publication

Written informed consent for publication of this case report and accompanying images was obtained from the patient. A copy of the written consent is available for review by the Editor of this journal.

## Availability of data and materials

The data are not publicly available due to issues of patient privacy. The datasets used during the current study are available from the corresponding author on reasonable request.

## Competing interests

The authors declare that they have no competing interests.

## Funding

No funding was obtained for this study.

## Author contribution statement

All authors listed have significantly contributed to the investigation, development and writing of this article.

We would like to thank Forte Science Communications (email: info@forte-science.co.jp) for English language editing.

## Declaration of competing interest

The authors declare that they have no known competing financial interests or personal relationships that could have appeared to influence the work reported in this paper.
